# Dual-band and dual-polarized reflective Fresnel zone plate design based on Fractal shape

**DOI:** 10.1038/s41598-023-33446-y

**Published:** 2023-04-17

**Authors:** Lusong Wei, Chao Ma, Xingyu Liu, Hongbin Ma, Yuting Wu, Zhanchun Fan, Jiangtao Huangfu

**Affiliations:** 1 Laboratory of Applied Research On Electromagnetics (ARE), College of Information Science and Electronic Engineering, Zhejiang University, Hangzhou, 310027 China; 2 Beijing Engineering Research Center of EMC and Antenna Measurement, Beijing, 100094 China

**Keywords:** Engineering, Electrical and electronic engineering

## Abstract

In this study, a dual-polarized and dual band reflective Fresnel zone plate with reconfigurable beam is proposed on the basis of fractal frequency selective surface (FSS) unit with nearly 360° phase tunability. Firstly, a new phase distribution calculation strategy based on Fresnel diffraction theory is proposed to improve the performance under certain scenarios like sparse arrays. Then, a novel fractal shape is put forward and applied to the design of the Fresnel zone plate. The introduction of the fractal structure makes the unit cell perform dual band, dual polarization and 309° phase tunability characteristics. Due to the self-symmetry of the unit cell, the proposed fractal Fresnel zone plate (FFZP) is capable of beam steering in ± 45° in both TE and TM incident waves. Besides, the proposed structure shows small performance degradation when it comes to oblique incidence up to 45°, which decreases the focal diameter ratio and profile of the proposed FFZP. The operating bandwidth of the FFZP can reach up to 700 MHz at X and Ku bands. It is applicable in a wide range of RF and microwave settings such as satellite and base station.

## Introduction

With the rapid development of the communication field, the corresponding communication equipment conditions are improved on a continued basis, which places higher requirements on transmission and receiving antenna, in terms of miniaturization, portability, multi-band, etc. As for antenna design, there has been research conducted on the multi-band and miniaturized high-performance antennas intended to replace multiple independent antennas. This is purposed to reduce the load of the installation platform, improve the reliability and electromagnetic compatibility of the system, and reduce the system cost, which has become an important objective set for modern antenna design.

In general, increasing the paths and resonant modes of surface current enables the antenna to operate at different frequencies while maintaining a relatively small size. In^1^, a coupled branch structure is incorporated into the antenna with an isolation distance to achieve multi-band characteristics, which is based on the characteristics of electromagnetic coupling between the branch and the antenna. In^2^, adjustment is made to the slotted structure of the antenna, therefore the surface current distribution at the edge of the slot for the antenna is changed, which enables the antenna to operate at different frequencies. In^3^, a similar design is proposed according to fractal theory. The introduction of fractal theory and structure inspires researchers to solve these knotty trade-off problems from a different perspective^4–9^.

Fractal theory is first put forward by Mandelbrot B.^10^. There are two distinct characteristics of the shapes based on Fractal theory: self-filling and self-similarity^11,12^. The former refers to filling the limited space from each dimension to the maximum extent. The latter refers to reducing or increasing the size of the fractal shape in any proportion while maintaining the irregularity of the overall shape in each similar dimension at basically the same level. Therefore, the unique properties of the fractal structure enable the fractal-based antenna/radome to perform well in size compression capability and multi-frequency broadband characteristics. Unlike traditional antennas, the miniaturization and multi-frequency characteristics of fractal antennas are requisite for the fractal structure, without any additional conditions.

Generally, lenses and arrays are used for beam forming and beam steering^13,14^. Arrays like reflectarrays and transmitarrays usually require high focal diameter ratio (F/D)^15–17^, which increases the profile of antennas especially when it comes to very large scale array. To lower the profile while making beam manipulation more flexible, planar Fresnel lens, known as Fresnel zone plate (FZP), has attracted a great deal of attention from scholars. In^18–20^, a 1-D FZP is implemented basing on the principle of electrically induced opacity and transparency. The FZPs proposed in ^21,22^ are based on optically controlled semiconductors. In^23,24^ bandpass filter theory is applied to the design of FZP for the development of 2D reconfigurable FZP.

Since Allain C. raised the concept of optical diffraction on fractals^25^, several researches have been conducted to focus on the combination of fractal theory and Fresnel diffraction. In^26^, Saavedra G. made theoretical derivation on fractal Fresnel zone plate. These limited researches are mainly focused on theoretical level and belong to optics field.

To meet the increasing demands for various applications, such as radar and satellite communications, a novel fractal structure is proposed herein to demonstrate a dual-band and dual-polarized Fresnel zone plate antenna. The proposed unit cell is capable of achieving a phase tunability of nearly up to 309° and shows small performance degradation under oblique incidence up to 45°, which increases the accuracy of beam steering while maintaining low focal diameter ratio of the whole antenna. Meanwhile, a new phase distribution calculation strategy based on Fresnel diffraction theory is proposed. Unlike traditional reflectarrays and transmitarrays, the reference point during the calculation can be arbitrary, thus making the proposed strategy more suitable when it comes to sparse array design. The rest of this paper is organized as follows. In “Principle” Section, the principle of the fractal Fresnel zone plate (FFZP) is introduced. “Design and simulation” Section elaborates on the analysis of the novel fractal unit cell and the practical biasing circuit. In “Fabrication and Measurement” Section, the performance of the proposed FFZP is verified through the beam steering experiment. In “Conclusion” Section , the potential of the proposed FFZP is analysed.

## Principle

**Figure 1:**
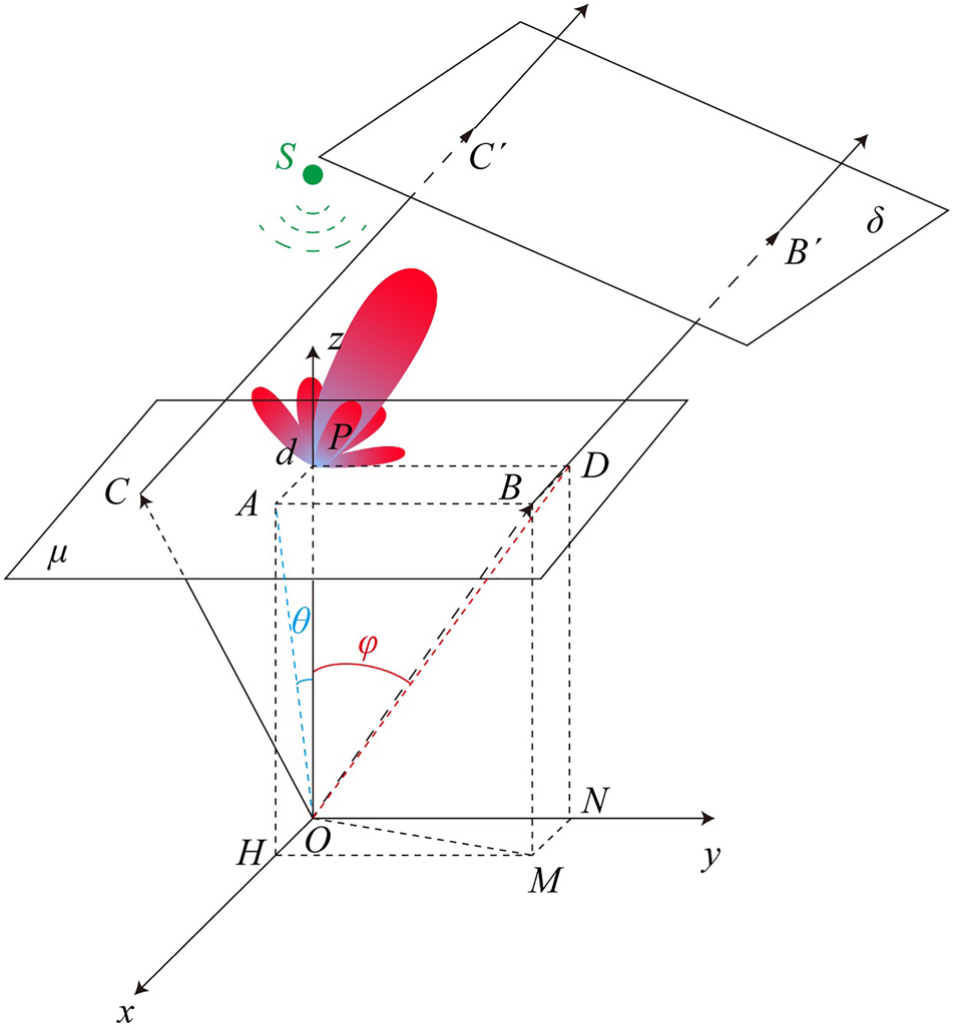
Principle of reflective Fresnel zone plate.

The principle of reconfigurable antenna based on Fresnel zone plate (FZP) is shown in Fig. 1. Herein, the reflected Fresnel zone plate is exemplified. In Fig.1, *d* represents the distance between the source and FZP diffractive strips, which is usually set to 3*λ* to ensure that the source is in Fresnel zone. When the equivalent spherical wave of the feeding antenna reaches the FZP, the FZP can be regarded as a secondary emission source according to Huygens principle. In this case, the far-field radiation of the entire antenna system can be obtained.

In addition, as per the basic requirement of the Fresnel region, the relative phase distribution can be calculated using the analytical geometry method based on geometrical optics. This method can avoid the computationally complex problems that are likely to arise from deducing trigonometric relations.

As shown in Fig. 1, the mirror of the feeding source *S* along the FZP plane *μ* is treated as the origin *O* of the coordinate system to better illustrate how the phase distribution of the FZP is analyzed. *OB* indicates the required beam direction. According to the trigonometric function, when the steering angle in direction *x* is ∠*POA* = *θ* and in direction *y* is ∠*POD* = *φ*, the coordinates of point *B* can be expressed as $\left(d\mathrm{tan}\theta ,d\mathrm{tan}\varphi ,d\right)$, and $\overrightarrow{OB}=(d\mathrm{tan}\theta ,d\mathrm{tan}\varphi ,d)$.

In order to simplify the phase difference calculation, *OB* is extended to *OB'*, and a vertical plane *δ* is established at *B'*. That is to say, *OB’* is the normal vector of plane *δ*. It is assumed that the extension magnification is *h*. In order to ensure that the area where the Fresnel zone plate is located is below the plane *δ*, *h* can be very large to simplify the calculation without affecting the final results. This is because it can be eliminated from the formula eventually. In this situation, $\overrightarrow {{OB^{^{\prime}} }} = h\overrightarrow {OB} = \left( {hd\tan \theta ,hd\tan \varphi ,hd} \right)$, so that the plane *δ* can be expressed as:

1 $$\begin{array}{*{20}c} {ax + by + cz + m = 0} \\ {\begin{array}{*{20}c} {a = hd\tan \theta } \\ {b = hd\tan \varphi } \\ \end{array} } \\ {\begin{array}{*{20}c} {c = hd} \\ {m = - \left[ {\left( {hd\tan \theta } \right)^{2} + \left( {hd\tan \varphi } \right)^{2} + \left( {hd} \right)^{2} } \right]} \\ \end{array} } \\ \end{array}$$

The distance between origin *O* and plane *δ* is expressed as:

2 $$d_{O\delta } = \sqrt {\left( {hd\tan \theta } \right)^{2} + \left( {hd\tan \varphi } \right)^{2} + \left( {hd} \right)^{2} }$$

Now, it is necessary to consider the other path *OC*, and $\overrightarrow{OC}=(x,y,d)$. Since path $\overrightarrow {{CC^{^{\prime}} }}$ is perpendicular to plane *δ*, the length of $\overrightarrow {{CC^{^{\prime}} }}$ is equal to the distance between point *C* and plane *δ*. It is assumed that point *C* is always below the plane *δ*, then

3 $$CC^{\prime} = \frac{{\left| {ax + by + cd + m} \right|}}{{\sqrt {\left( {hd\tan \theta } \right)^{2} + \left( {hd\tan \varphi } \right)^{2} + \left( {hd} \right)^{2} } }} = \frac{{ - m - \left( {ax + by + cd} \right)}}{{\sqrt {\left( {hd\tan \theta } \right)^{2} + \left( {hd\tan \varphi } \right)^{2} + \left( {hd} \right)^{2} } }}$$

The distance from point *O* via *C* to *C'* is expressed as:

4 $$d_{O\delta ^{\prime}} = \sqrt {x^{2} + y^{2} + d^{2} } + \frac{{ - m - \left( {ax + by + cd} \right)}}{{\sqrt {\left( {hd\tan \theta } \right)^{2} + \left( {hd\tan \varphi } \right)^{2} + \left( {hd} \right)^{2} } }}$$

Due to mirroring effect, the phase between point *S* and point *O* is opposite. Thus, the phase difference between the two paths ${d}_{S\delta }$ and ${d}_{S{\delta }^{^{\prime}}}$ is expressed as:

5 $$\begin{aligned} \Delta \varphi = k\left( {d_{{O\delta^{\prime}}} - d_{O\delta } } \right) + {\pi } = & k\left[ {\sqrt {x^{2} + y^{2} + d^{2} } + \frac{{ - m - \left( {ax + by + cd} \right)}}{{\sqrt {\left( {hd\tan \theta } \right)^{2} + \left( {hd\tan \varphi } \right)^{2} + \left( {hd} \right)^{2} } }}} \right] - k\sqrt {\left( {hd\tan \theta } \right)^{2} + \left( {hd\tan \varphi } \right)^{2} + \left( {hd} \right)^{2} } + \pi \\ = & k\left [ {\sqrt {x^{2} + y^{2} + d^{2} } - \frac{{d\tan \theta x + d\tan \varphi y + d^{2} }}{{\sqrt {\left( {d\tan \theta } \right)^{2} + \left( {d\tan \varphi } \right)^{2} + \left( d \right)^{2} } }}} \right] + \pi \\ \end{aligned}$$

According to the phase difference $\Delta \varphi$, the phase distribution of the secondary emission source on the Fresnel zone plate can be calculated. On this basis, phase compensation can be performed on the spherical wave radiated from the feeding source. In this way, beam steering is achieved. In this section, the phase difference calculation is analyzed by geometrical optics. In most cases, this calculation strategy is as same as the traditional reflectarrays. The novelty of the proposed method is that the reference point can be any point while the center point is always set as the reference point in reflectarrays. Because of the arbitrariness of the selection of the reference point, the proposed strategy is more suitable when it comes to sparse array design.

## Design and simulation

Herein, the dual polarized and dual band reconfigurable reflective Fresnel zone plate is designed in line with the principle as mentioned above and the inherent property of fractal theory.

According to fractal theory, the process of fractal generation can be conducted through a series of affine transformation, which is also referred to as iterative function system (IFS). When (*x*, *y*) is assumed to be a point in the initial graph, (*x*_1_, *y*_1_) is the affine transformation of (*x*, *y*) and it can be expressed as:

6 $$w\left( {\begin{array}{*{20}c} {x_{1} } \\ {y_{1} } \\ \end{array} } \right) = \left( {\begin{array}{*{20}c} {r\cos \Psi } & { - s\sin \xi } \\ {r\sin \Psi } & {s\cos \xi } \\ \end{array} } \right)\left( {\begin{array}{*{20}c} x \\ y \\ \end{array} } \right) + \left( {\begin{array}{*{20}c} {x_{0} } \\ {y_{0} } \\ \end{array} } \right)$$

where *r* and *s* represent the scaling ratios, $\Psi$ and $\xi$ denote the rotational changes, and *x*_0_ and *y*_0_ refer to the translation changes in directions *x* and *y*, respectively.

**Figure 2:**
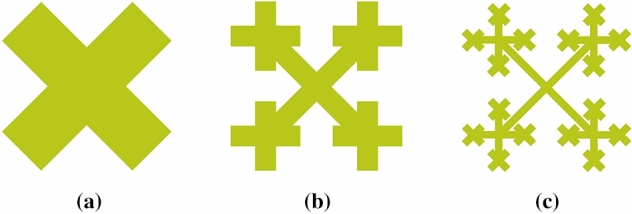
Fractal iterative process (***a***) *n* = 0; (***b***) *n* = 1; (***c***) *n* = 2.

In Figure 2 a novel fractal structure is proposed. The zero-order fractal of the structure is a letter X and defined as structure letter X (SLX). The first-order fractal is formed by scaling the zero-order fractal shape to 1/*r* in comparison with itself. Upon a 45° rotation, it is translated to form four small cross shapes. This layer is defined as structure cross shape (SCS) layer. Then, the SLX layer is used to connect the SCS layer, thus forming Fractal tree. The iterative process is almost unchanged in case of higher order fractal shapes.

Let *G* represent the initial graph and *W*(*G*) represent the affine transformation performed on the original graph *G*, then the fractal iteration process can be expressed as:

7 $$\begin{array}{*{20}c} {W_{1} \left( G \right) = \left( {\begin{array}{*{20}c} {r\cos 45^\circ } & { - r\sin 45^\circ } \\ {r\sin 45^\circ } & {r\cos 45^\circ } \\ \end{array} } \right)\left( {\begin{array}{*{20}c} x \\ y \\ \end{array} } \right) + \left( {\begin{array}{*{20}c} {x_{0} } \\ {y_{0} } \\ \end{array} } \right)} \\ {W_{2} \left( G \right) = W\left( {W_{1} \left( G \right)} \right)} \\ {\begin{array}{*{20}c} {W_{n} \left( G \right) = W\left( {W_{n - 1} \left( G \right)} \right)} \\ {W_{fractal - tree} \left( G \right) = G \cup W_{1} \left( G \right) \cup W\left( G \right) \cup \cdots \cup W_{n} \left( G \right)} \\ \end{array} } \\ \end{array}$$

As shown in Figure 2. each iterative process leads to the generation of four new sub-shapes. Therefore, the fractal dimension *Ds* of the proposed fractal tree structure is expressed as:

8 $${\text{Ds}} = \mathop {\lim }\limits_{r \to 0} \frac{{\log N\left( {G,r} \right)}}{{\log \left( {1/r} \right)}} = \frac{\log 4}{{\log \left( {1/r} \right)}}$$

**Figure 3:**
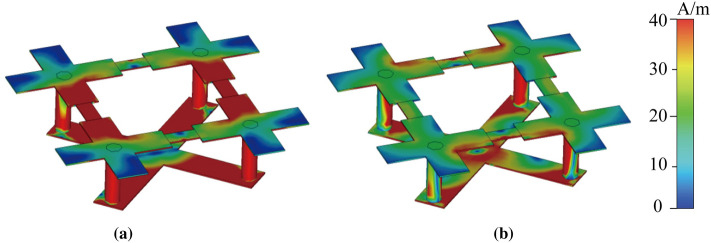
Surface current distribution when *C*_v_ = 0.08pF at (**a**) 9.5 GHz (**b**) 14.5 GHz.

The first-order of the proposed fractal structure is taken as the unit cell of the FFZP. In order to analyse its electrical characteristic, a practical model based on a certain set of parameters was established on CST^TM^ microwave studio, and the surface current distribution at different frequencies is simulated, the results of which are shown in Fig. 3.

The connected SLX and SCS layer tends to have a greater equivalent electric length, which means higher inductance, thus leading to the low-frequency-resonance. By comparison, the SLX itself has a shorter equivalent electric length which leads to the high-frequency-resonance.

Then, a step further is taken for its application. Figure 3 illustrates the structure of the designed unit cell in detail. Rogers 4003C is treated as the substrate, whose relative dielectric constant and loss tangent are 3.55 and 0.0027, respectively. Another two substrates are sandwiched by four metallic sheets.

Showing 4 cross shapes, the SCS layer of metallic sheet is connected by 4 varactors (MACOM MAVR-011020-1411), whose equivalent circuit is comprised of a series 0.05nH inductance, and a 2-ohm resistance and a capacitance varying from 0.025 to 0.19pF via reversed DC voltages varying from 0 to 20V. The SLX layer is connected to the SCS layer with 4 buried holes, thus forming a fractal tree structure. The optimized dimensions of the structure are shown in Table 1.

**Table 1:** Dimensions of the proposed unit cell (Unit: mm).

Parameter	Value	Parameter	Value	Parameter	Value	Parameter	Value
*a*_1_	2.8	*b*_1_	2.8	*d*_1_	0.4	*c*_1_	2.4
*a*_2_	0.5	*b*_2_	0.4	*d*_2_	0.3	*c*_2_	2.55
*a*_3_	0.8	*b*_3_	0.4	*d*_3_	0.3	*c*_3_	0.3
*a*_4_	3.2	*b*_4_	0.2	*d*_4_	0.8	*h*_1_	1.5
*a*_5_	5.6	*b*_5_	0.8	*α*	90°	*h*_2_	0.2
*a*_6_	8	*b*_6_	8	*β*	60°	*h*_3_	0.8

**Figure 4:**
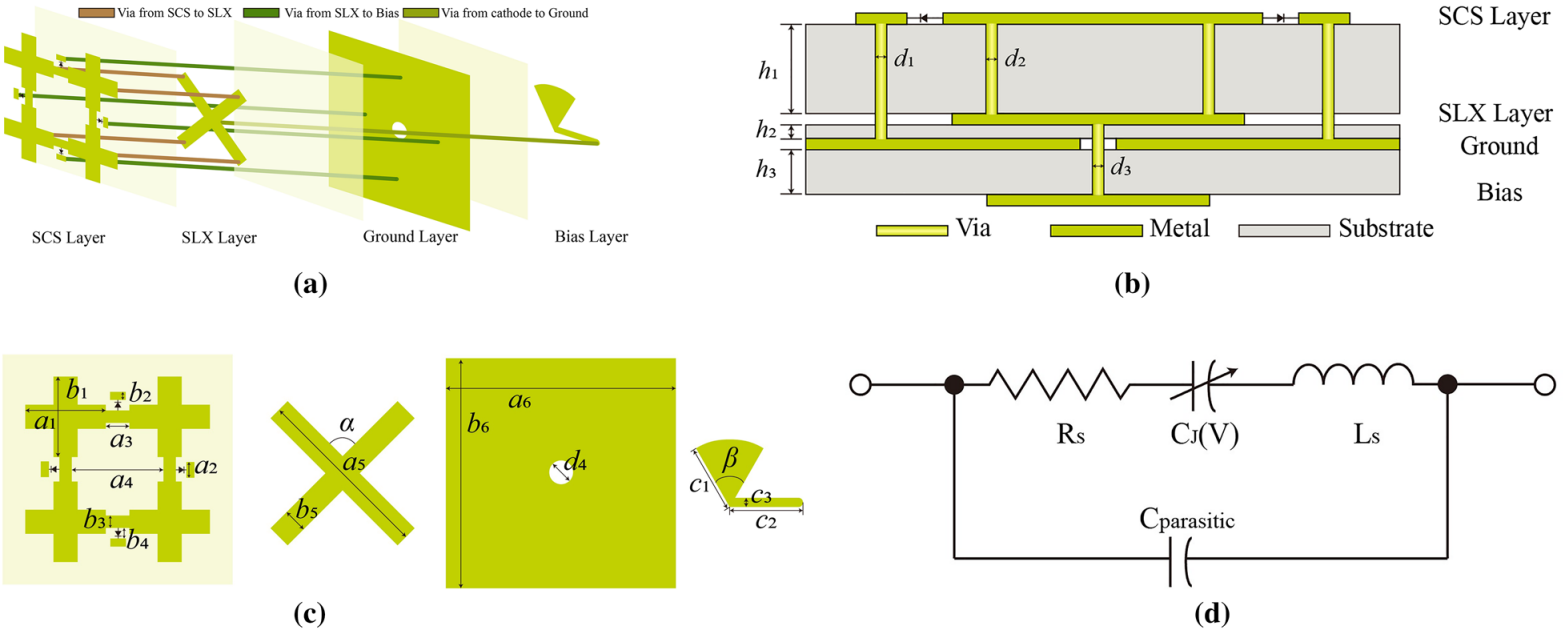
(**a**) Schematic diagram (**b**) Profile of layered structure (**c**) Parameter statement of the proposed unit cell (**d**) Equivalent model of the varactor.

The third and fourth layers are ground plane and bias layer, respectively. The biasing circuit plays a significant role in unit cell design for practical application. As shown in Fig. 4, a practical biasing architecture is proposed to enable the simultaneous control of the four varactor diodes. One DC via, which connects SLX and the bias line, with clearance from the ground is adopted at the equivalent zero-electric-field point, i.e., the center of the patch. Bounded at the back of the ground plane, a thin Rogers 4003C substrate layer is introduced to facilitate biasing routings. Also, a 60° open-ended radial stub printed on the 4003C is used to choke the RF signals. Moreover, the radial stub with a radius of 2.4 mm along with the transmission line *c*_2_=2.55mm, *c*_3_=0.3mm is used to apply an equivalent open-circuit load on the patch.

**Figure 5:**
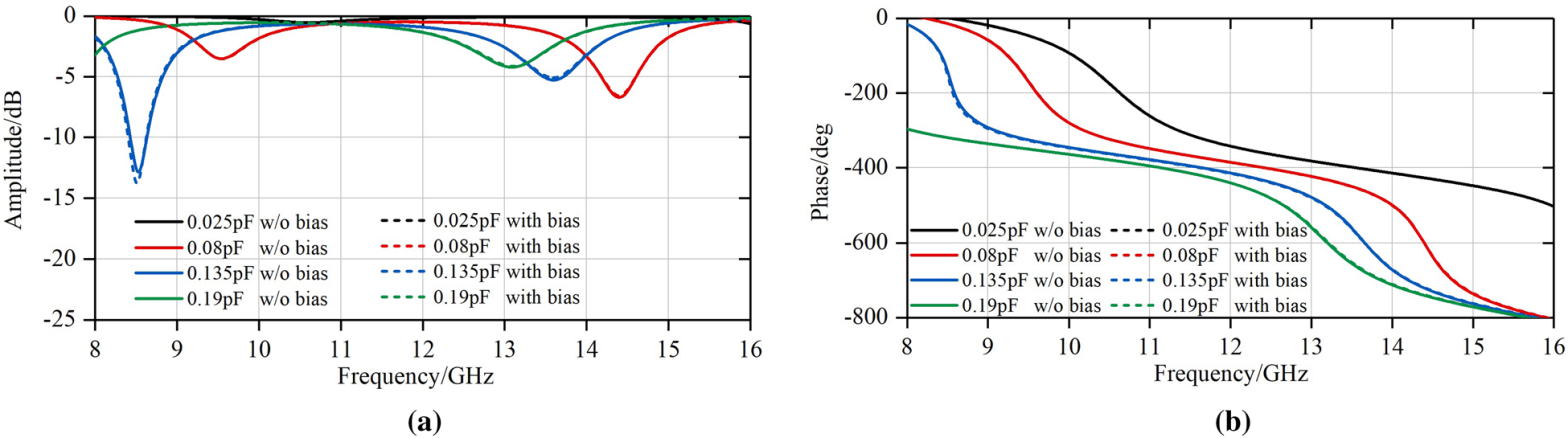
Comparison of simulated reflection coefficients *S*_11_ at TE incident wave whether there exists bias layer or not. (**a**) Amplitude. (**b**) Phase.

The reflection performance with or without biasing was compared, as shown in Fig. 5. From this figure, it can be observed that the biasing circuits make little difference to reflection loss and phase. The phase difference remains basically unchanged across the frequency band after dc biasing. These results evidence the excellent performance of the proposed biasing design.

**Figure 6:**
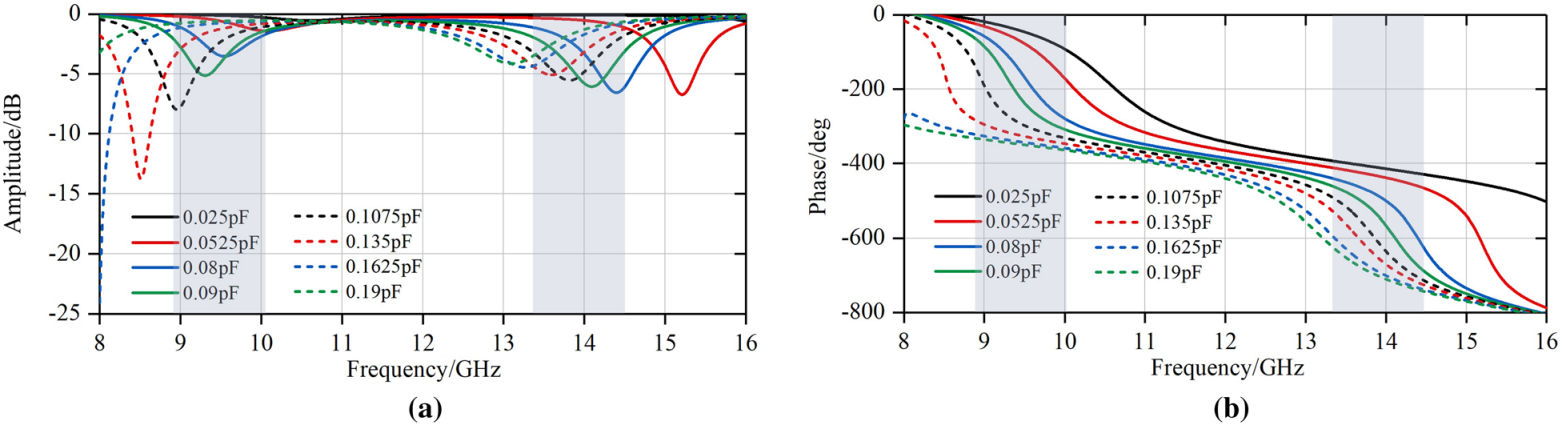
Simulated reflection coefficients *S*_11_ at TE and TM incident wave. (**a**) Amplitude. (**b**) Phase.

Since the unit cell shown in Fig. 4 is symmetric for *x*- or *y*-polarized incident wave, this design is supposed to be capable of dual linear polarization, as shown in Fig. 6. The proposed unit cell is simulated in CST^TM^ microwave studio.

It can be observed from above that there are two operating frequency bands (X and Ku band). When *C*_v_ varies from 0.025 to 0.19pF, there is a shift in resonant frequency, with the corresponding phase tunability reaching up to 320° both in X band and Ku band according to similar amplitude standard in ^24,27^. If the phase tunability is practically more important than insertion loss, the operating bandwidth can approach 1GHz with phase tunability being equal to 300°. And from Fig. 6, the reflection magnitude response S11 shows higher phase tunability in X band than Ku band. The reasons are as follows. The SLX is connected to SCS with 4 vias. By placing the two layers on the two sides of the substrate, a plane-parallel capacitor is obtained to improve the Q factor of the structure and enhance the resonance of the structure, thus extending the phase tunability.

**Figure 7:**
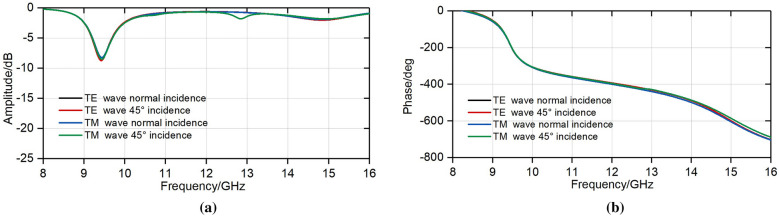
Simulated reflection coefficients *S*_11_ at oblique incidence when *C*_v_ = 0.1pF. (**a**) Amplitude. (**b**) Phase.

Meanwhile, the proposed structure shows small performance degradation when it comes to oblique incidence. According to the curves in Fig 7, phase of oblique incident wave shows slight change compared with normal incident wave, which indicates that the focal diameter ratio of the proposed FFZP can be decreased at large scale.

## Fabrication and measurement

**Figure 8:**
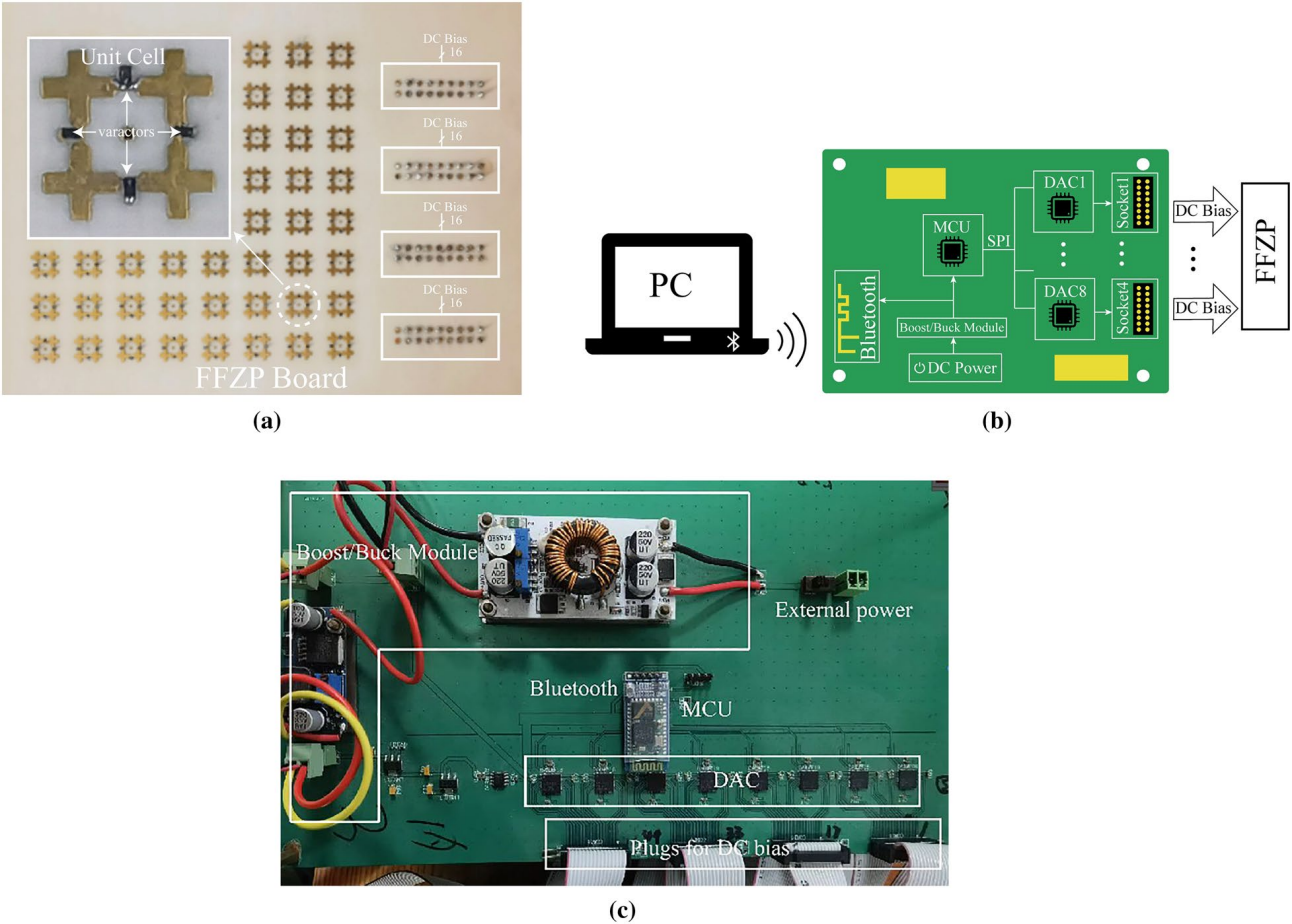
(**a**) Fabricated FFZP board; (**b**) Schematic diagram of the control circuit; (**c**) Fabricated control board.

To verify the proposed unit cell, a 2.5-mm-thick low-profile FFZP was fabricated with the same substrates and dimensions as discussed in previous sections. It is illustrated in Fig. 8.

As shown in Fig. 8a, the fabricated FFZP consists of 8×8 fractal unit cells, covering an area of 64mm*64mm except for the DC bias sockets considering of costs. The unit cell is soldered with 4 varactors. There are four sockets deployed at the right side of the FFZP board to control the unit cells separately via the control board shown in Fig. 8b. At first, the required voltage for each unit cell is calculated in PC and the information is transmitted to MCU (STM8S105K4) through the Bluetooth module (BlueCore4-Ext). Then, MCU selects the target DAC chip (DAC7718) by selecting the appropriate CS line, before the processed voltage data are written to the target output terminal of the target DAC chip. 8 DAC chips offer 64 paths of voltages for the FFZP board.

To measure the reflection performance of the proposed unit cell, a rectangular waveguide was used. This test method relies on the mirror image to characterize the unit cell in an equivalent infinite periodic arrangement, the results of which are shown below.

It can be seen from above that when the DC voltage varies from 1.5V to 15.5V, two operating frequency bands emerge, namely, X band (9.1GHz to 9.8GHz in TE mode and 9.08GHz to 10.12GHz in TM mode) and Ku band (13.8GHz to 14.5GHz both in TE and TM mode), with the maximum tunability reaching up to 309°. It demonstrates the feasibility of the proposed unit cell and control board.

**Figure 9:**
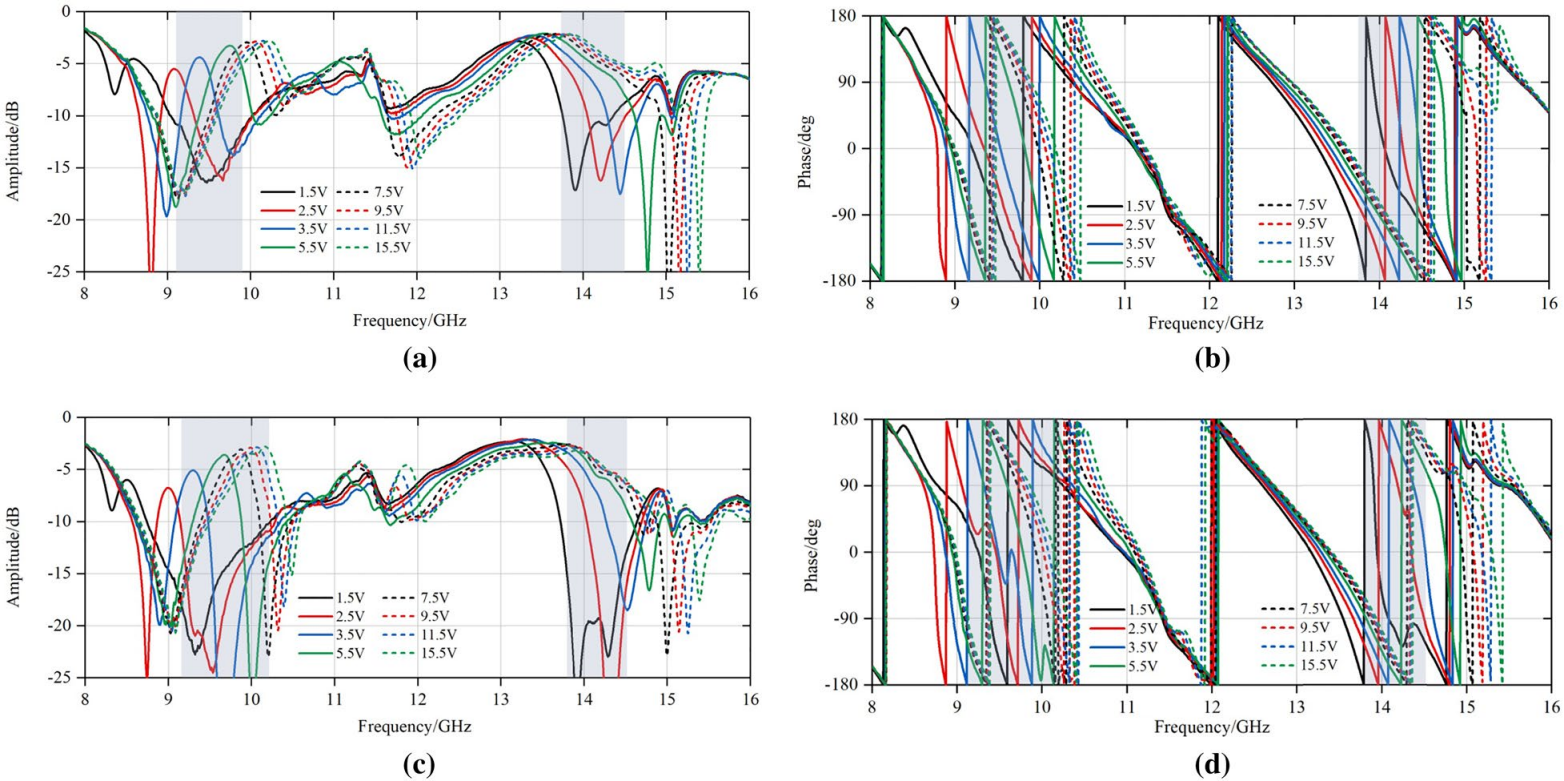
Reflection measurement of the fabricated Experiment. (**a**) Amplitude in TE mode. (**b**) Amplitude in TM mode. (**c**) Phase in TE mode. (**d**) Phase in TM mode.

From Fig. 9, the response of the prototype presents higher losses than the theoretical results in Fig. 6. The losses mainly come from the package and soldering of the varactor diodes. These factors become more sensitive above X band, thus bringing higher losses than theoretical analysis.

**Figure 10:**
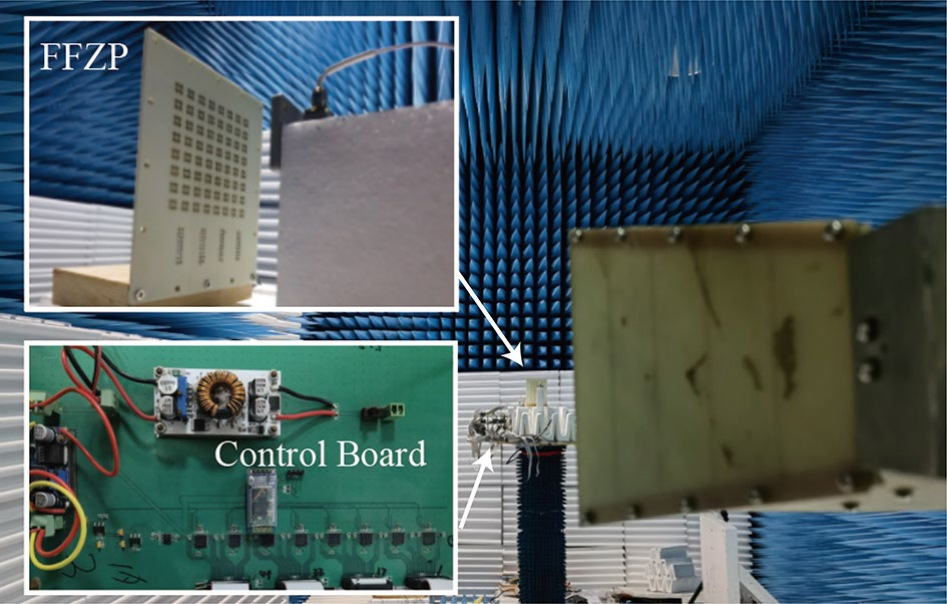
Experimental setup for the beam-steering measurement.

Then, the beam-steering measurement environment was established in an anechoic chamber as shown in Fig. 10. A waveguide coaxial connector (HD-120WCAS) operating from 9GHz to 14.5GHz was adopted as the feed source with a 40mm gap in front of the FFZP, which indicates that the focal diameter (*f*/D) is 0.625. A dual ridged horn antenna (HD-1018DRHA) was treated as the receiving antenna. Then, the network analyzer (R&S ZNB20) was employed to measure the transmission amplitude and phase at the require frequency band (9–14.5GHz). According to the reciprocity principle, the radiation pattern of the FFZP can be identified.

**Figure 11:**
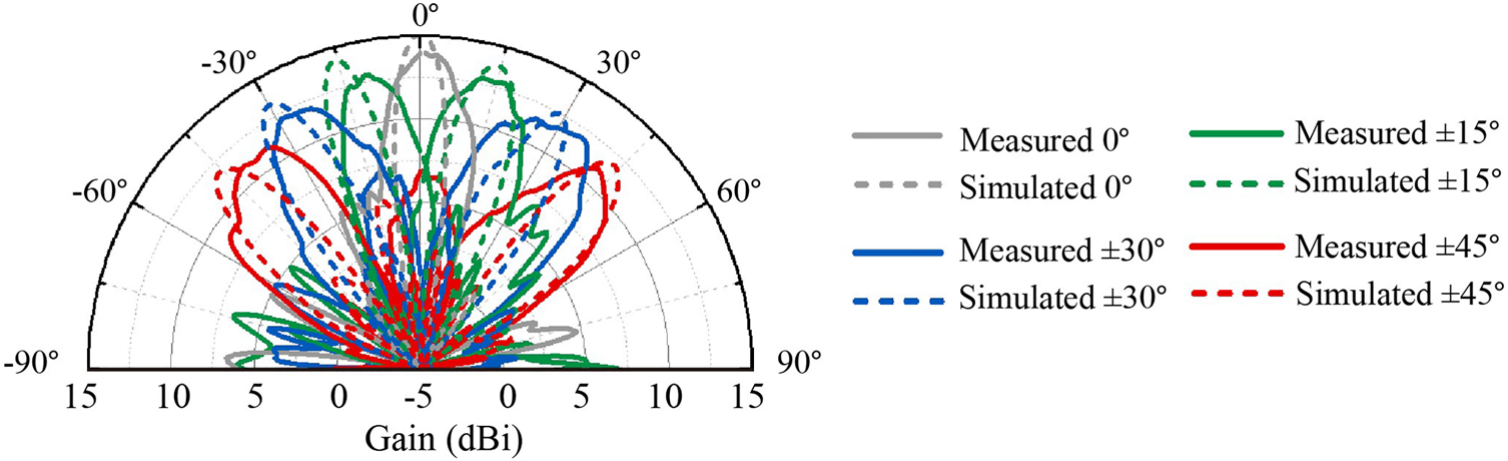
Comparison between simulated and measured beam steering results at 9.75 GHz (TE incident wave).

**Figure 12:**
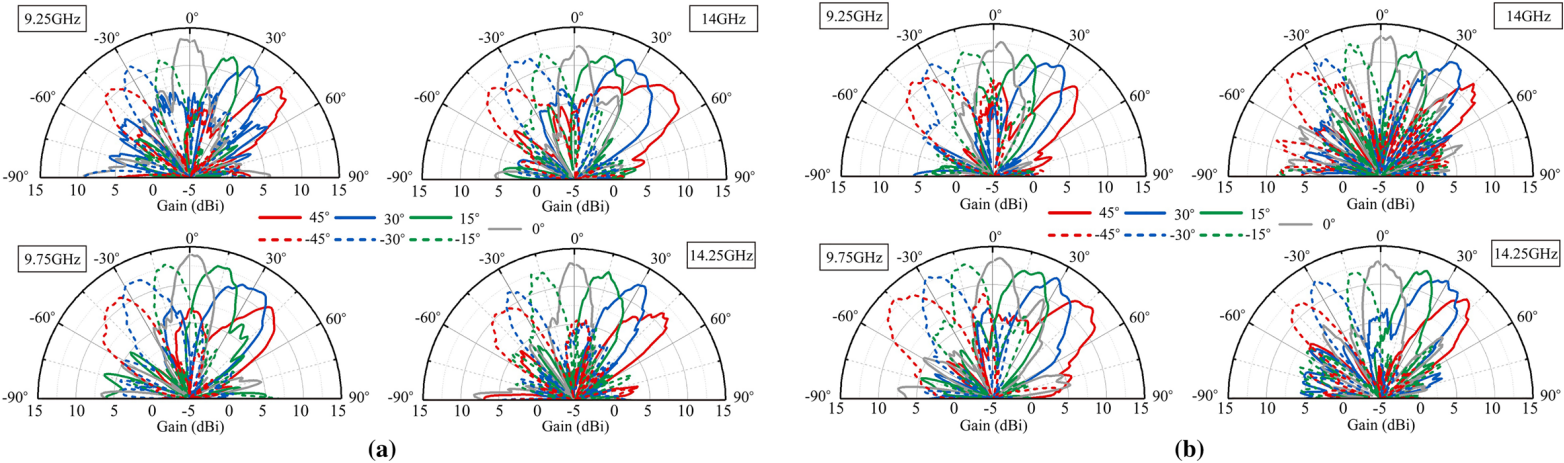
Beam steering results at different frequencies at (**a**) TE incident wave. (**b**) TM incident wave.

The scenario at 9.75GHz under TE incident wave is simulated among numerous scenarios as a verification of the design. The simulated beam steering results compared with the measured beam steering results is shown in Fig 11. The measured beam show basically the same as the simulated beam which verifies the design. Then the detailed measured radiation patterns at other scenarios are shown in Fig. 12. It can be seen from the figure that the proposed FFZP shows dual polarized and dual band characteristics, which is consistent with the simulation results. The beam steering performance can reach up to ±45°. The gain of the proposed FFZP can reach a maximum of 13.7dBi and achieve an improvement of at least 2.5dBi in the dual frequency band compared to the original waveguide coaxial connector. Moreover, the gain change of the proposed FFZP during the beam scanning process is around 2dBi, which shows good gain robustness.

Table 2 lists the performance of the proposed FFZP compared with other literatures. It can be concluded that the proposed antenna shows higher flexibility in beam control with smaller size and lower *f*/D.

**Table 2:** Comparison of the proposed structure with other literatures.

Ref	Bands of operation	Unit cell size [mm]	*f*/D	Beam steering control	Phase tunability	Polarization
This work	X/Ku	8	0.625	± 45°	309°	Dual-linear
^15^	X/Ku	8	0.8	No	180°	Dual-linear Dual-circular
^16^	X/Ku	14	4	± 24°	300°	Circular
^17^	X/Ku	15	1.3	No	360°	Dual-linear

And finally, despite the dual band beam steering characteristics exhibited by the proposed FFZP, it remains applicable for multiple frequency bands by increasing the orders of the fractal shapes. Herein, a new solution to the design of multiple frequency band and miniaturized antenna radome is put forward, which sheds light on diverse microwave and even light applications.

## Conclusion

In this study, a dual-polarized and dual band reflective Fresnel zone plate with reconfigurable beam was proposed on the basis of fractal FSS unit with 309° phase tunability. Unlike traditional reflectarrays, a new phase distribution calculation strategy based on Fresnel diffraction theory was proposed to improve performance under certain scenarios like sparse arrays. Due to the self-filling, self-similarity, self-symmetry characteristic of the fractal shape, the proposed FFZP is capable of beam steering at ±45° in both TE and TM incident waves at X-band and Ku-band, while expanding to other frequency bands without highly complex design procedure. Besides, the unit cell of the proposed structure shows small performance degradation when it comes to oblique incidence up to 45°, which decreases the focal diameter ratio and profile of the proposed fractal Fresnel zone plate at large scale. The design has great application potential in radar detection, satellite and wireless communications.

## Data availability

The data produced and analyzed during the current study are available from the corresponding author on reasonable request.
